# A comparison of weighted countermovement jumps loading modes using wearable accelerometers

**DOI:** 10.17159/2078-516X/2024/v36i1a16929

**Published:** 2024-09-15

**Authors:** V Radulovic, D Kwong, A Green

**Affiliations:** Department of Sport and Movement Studies, Faculty of Health Sciences, University of Johannesburg, South Africa

**Keywords:** mechanical power, jump testing, modes of loading

## Abstract

**Background:**

The countermovement jump (CMJ) is an integral part of force and velocity profiling; a movement that is regularly implemented in training protocols and testing of athletic performance. Adding external loads to CMJs may have an added benefit for assessing gains in power and, in turn, monitoring progressive development. However, these added loads can displace the centre of mass of individuals, which may alter jump kinetics.

**Objectives:**

The study aimed to evaluate kinetics across various incremental modes of loading (barbell, trapezius barbell, and dumbbell) CMJs.

**Methods:**

Thirty-two male athletes (age: 19±2 years; height: 1.86±0.06 m, mass: 90.4±5.3 kg) completed three weighted CMJs (20, 40, 60 kg) across three bar-type modes of loading (barbell, trapezius barbell, and dumbbell). Jump metrics were measured using a wearable accelerometer. A repeated measures ANOVA was used to compare jump metrics (p<0.05).

**Results:**

The results indicated changes in jump kinetics as added loads increased across all bar-type jump modes (p<0.001). Additionally, jump modes yielded different jump kinetics (p<0.001). Specifically, dumbbell CMJs produced the greatest force (2559 ± 462 N) and power (4861±1632 W) outputs. In contrast, the trapezius barbell consistently produced significantly (p<0.001) higher velocity (2.52±0.44 m.s^−1^) and acceleration (12.59±4.49 m.s^−2^), with the barbell never producing the highest kinetic metrics. The athletes’ ranges of movement and comfort loading levels during the CMJs may be influential factors affecting vertical jump output metrics.

**Conclusion:**

Overall, jump kinetics were altered by loads and jump types. Practically, different loading methods may target distinct jump variables allowing for individualised training programs specific for the athletes’ needs.

The countermovement jump (CMJ) is defined as a power movement, in which the athlete relies on the muscles of the lower extremity to propel the body upwards into the air in an explosive manner, from squatting down to a self-determined depth. The simple CMJ implements a stretch-shortening cycle, which involves a stretching of the muscle-tendon unit, immediately followed by a rapid shortening of the muscle unit, allowing for a quick explosive movement.[1, 2] As a common method of assessing the athletic ability of individuals, the average CMJ variables are more sensitive than that of other standalone lower extremity power movements in tracking the super-compensatory behaviour of the legs and lower body.[1] Indeed, numerous studies have indicated the importance of CMJ testing[2, 3] and training interventions for improved physical performance.[4, 5] Performance of CMJ has been related to general sports tasks, namely, sprint and change of direction abilities.[5] Additionally, CMJ metrics can be used to track and monitor physical development.[2]

Loaded CMJs, where additional external load is applied to the athlete, are a valuable tool for both research and training. While centre of mass (CoM) displacement is the major output of vertical jumps, loaded CMJs provide insight into how CoM displacement and the movement's degrees of freedom (DoF) affect jump performance.[6] There are different modes of loading a CMJ.[7, 8] Loaded CMJs directly affect CoM displacement by varying the load parameters along the vertical axis. These variations also impact the DoF by either increasing or restricting an individual’s freedom of movement and, subsequently, the force-velocity-power relationship. The most common loading mode in CMJ is completed by placing a straight barbell resting on the shoulders. However, individuals may experience discomfort during the barbell CMJ.[9] Loading in more natural positions such as a trapezius barbell or dumbbells may be suitable alternatives.[10] Trapezius barbells require athletes to step inside of a hexagonal-shaped frame to grip the bar suspended at a mid-thigh height. Dumbbells, held independently in each hand, offer similar positioning and higher freedom of movement.

Loading kinetics play a role in the probability that individuals shall be able to chain movements together in an effective manner, which influences the jump metrics and mechanics of the movement. The different modes of loading yield different outputs, as they are influenced by CoM, DoF and skeletal muscle tension throughout the movement patterns.[11] Overall, the barbell CMJ may impose greater movement restrictions and in turn, reduce jumping kinetics. Implementing a trapezius barbell allows individuals to jump up with a relatively higher velocity than that of a straight barbell.[10] Furthermore, the addition of a slight arm swing when implementing dumbbell CMJs can alter kinetic outputs produced by athletes, subsequently increasing the power produced during the movement.[7] Interestingly, comfort and freedom of movement associated with different loading methods appear to have a greater impact on jump metrics than CoM displacement itself.[11]

Assessing the different measuring methods is integral to understanding the most appropriate and effective way of testing one’s CMJ metrics. There are certain devices that are extremely precise and capable of attaining highly accurate results. Specifically, most kinetic assessments of CMJ utilise force plates and linear transducers. However, these devices are expensive and are rarely incorporated into daily testing and training practices.[2] This is because devices like force plates tend to be quite immobile, and the set-up procedure makes them only work in laboratory conditions.[1] Conversely, there are wearable devices that are mobile and easy to set up but may not be as precise, primarily due to device sensitivity and placement during the various modes of CMJs. There are, however, accelerometers that can produce reliable and accurate data.[1] Accelerometers are mobile alternatives that allows for an individual to take the testing out of the laboratory onto the playing field, making the collection of data a much easier process.[1] Additionally, they are also a very cost-effective alternative to other data collection devices, allowing for greater accessibility amongst both coaches and sporting institutions that may otherwise not be able to record jump data regularly.[1]

Overall, CMJ testing uses a standard straight barbell and force plate setup. Furthermore, a trapezius barbell has been compared to the standard weighted CMJ protocol. However, a comparison between barbell, trapezius barbell and dumbbell modes of added loads has not been evaluated. The study aimed to evaluate the kinetics of weighted CMJs under various bar-type modes and added loads using mobile, wearable accelerometers.

## Methods

### Participants

A quantitative cross-over study consisting of 32 athletic male participants (age: 19±2 years, height: 1.86±0.06 m, mass: 90.4±5.3 kg) was conducted. All participants were free from any injuries for at least three months, had resistance training experience and provided signed informed consent prior to the study. Institutional ethical approval was obtained (REC-1107-2021).

### Testing procedures

Data were collected over four weeks in the pre-competition phase at a similar time (06:00–09:00) to avoid diurnal effects. A standardised researcher-led warm-up was completed. Specifically, each session started with static and dynamic stretching followed by low-intensity (4/10 RPE) cycling on a stationary cycle ergometer for five minutes. Incorporated into their warm-up, participants performed familiarisation jump tests for each jump mode. These tests were performed using a plastic dowel on the shoulders (barbell); at waist height (trapezius barbell); and with freely moving arms (dumbbell). Day one started with a standardised warm-up, followed by the unweighted CMJ (as part of the warm-up) and one loaded jumping variation. The jump test sequence was randomised on each day, with sequential loading. That is, jumps using the added loads of 20 kg were completed prior to 40 kg and 60 kg attempts. Day two, at least 48 hours after the first session, began with the same standardised warm-up, following which the participants completed the remaining two jump variations.

### Anthropometrics

Body stature was measured to the nearest 0.1 cm (stadiometer-model: Seca 213), and body mass to the nearest 0.01 kg (Seca Robusta 813).

### Countermovement Jumps

The standardised procedures for the CMJs were implemented, as previously described.[12, 13] Athletes stood with feet shoulder-width apart, descended into a self-determined squatted position (with at least 90 degrees knee flexion) and attempted to attain maximal height while maintaining a full leg extension.[13] The CMJ modes were varied as follows: the barbell CMJ was conducted with a straight barbell placed on the shoulder girdle behind the neck; trapezius barbell CMJ, where trapezius barbell was suspended at the mid-thigh and held in the hands; and the dumbbell CMJ, where participants held dumbbells suspended at thigh level with arms held in the fundamental position. The added loads (20, 40 and 60 kg) were placed onto the barbell, trapezius barbell and dumbbells. In the case of the dumbbells, the total load was divided into equal pairs, one held in each hand. Participants completed at least three attempts for every jump mode and load (nine jumps per mode) and were permitted to rest for at least 90 seconds between jump efforts. Accelerometers (PUSH band 2.0, PUSH, Toronto, Canada) were used to measure the jump kinetics (force, velocity, acceleration and power).[14] The device was placed on the barbell and trapezius barbell for the respective jumps and placed around the waist of the individuals when participants performed dumbbell CMJs. The best jump attempts (highest peak power) were extracted for analysis.

### Data analysis

All data were tested for normality amongst the athletes using the Shapiro-Wilk test. All data are presented as mean±SD. Jump kinetics (force, velocity, acceleration, and power) were compared across the various jump modes (bar-type) and loads (additional weights), using repeated measures ANOVA and, where applicable, Bonferroni *post-hoc* tests. All statistical analyses were conducted in Statistical Package for the Social Sciences version 27 (SPSS, IBM) with a significance level of p<0.05.

## Results

**Fig. 1:**
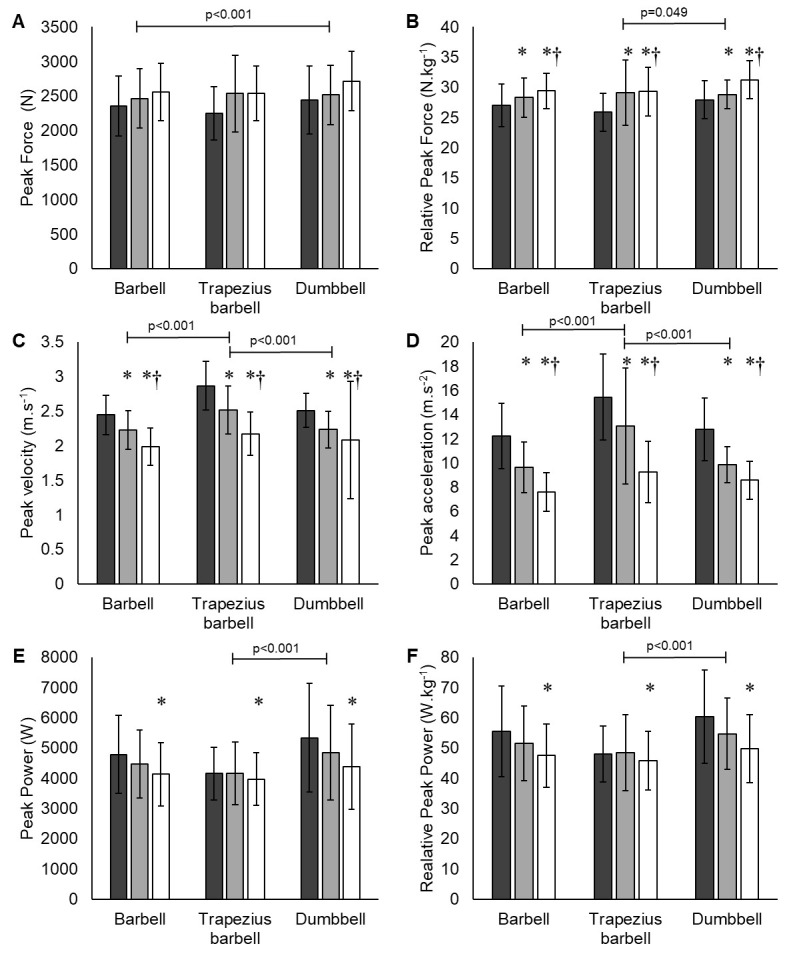
A comparison of countermovement jump kinetics between various external weighted loads and bar-type jump modes. (A) Peak Force; (B) Relative Peak Force; (C) Peak Velocity; (D) Peak Acceleration; (E) Peak Power; (F) Relative Peak Power. Dark grey bars indicate 20 kg, light grey bars indicate 40 kg and white bars indicate 60 kg loads. * Significantly different from 20 kg load; † significantly different from 40 kg load. Horizontal bars indicate significant differences across bar-type jump modes (p<0.05).

The summary kinetic jump variables are depicted in Table 1 and Figure 1. The average peak force increased as the load of the weighted jumps increased. Figure 1A shows a significant difference (p<0.001) across the jump loads. Specifically, jumps with 20 kg (2351±435 N) produced significantly lower force outputs than jumps with 60 kg (2605±416 N). In terms of bar-type jumps, there was no significant difference in force production across the jumps between the trapezius barbell (2442±468 N) and the barbell (2460±430 N). In contrast, the dumbbell CMJ had a significantly higher output (2559±462 N) than the other two jump modes (Table 1). However, significant differences between bar-type jumps are noted in Figure 1B when assessing relative forces. Specifically, jump mode differences were noted between the dumbbell (29.4±2.9 N.kg^−1^), the barbell (28.3±3.3 N.kg^−1^) and trapezius barbell (28.1±4.2 N.kg^−1^). Overall, there were no statistical interactions between the bar-type jumps and weighted loads (p=0.844).

Average peak velocities systematically decreased as the loads increased, all with recorded significant differences (p<0.001) (Figure 1 C). The average peak velocity of the trapezius barbell (2.52±0.44 m.s^−1^) CMJ is shown to be significantly higher (p<0.001) than that of both barbell (2.22±0.33 m.s^−1^), and dumbbell (2.28±0.56 m.s^−1^). No significant interaction was detected between bar-type jump modes and weighted loads of peak velocity (p=0.324). In line with the decrease in velocity with increased load, acceleration declined with increasing jump loads (Table 1). Similarly, the trapezius barbell acceleration (12.59±4.49 m.s^−2^) was significantly greater than (p<0.001) barbell (9.81±2.88 m.s^−2^) and dumbbell (10.41±2.63 m.s^−2^) acceleration (Figure 1D). No significant weighted load and bar-type jump mode interaction was noted for acceleration (p=0.100).

The average peak power decreased as the loads of the weighted jumps increased (Table 1). A significant difference (p<0.001) was recorded across the jump loads. Particularly, a significantly lower output was recorded for the 60 kg jump (4168±1136 W) in relation to the 20 kg jump (4765±1440 W). Additionally, there was a significant difference (p<0.001) between bar-type jump modes, with trapezius barbell (4104±927 W) showing the lowest average peak power of the three jumps (Figure 1E). However, no significant interaction was noted between the bar-type jump mode and weighted load for average peak power (p=0.545).

There was a significant difference between both bar-type jump modes and weighted loads in average peak relative power (p<0.001). Specifically, the dumbbell CMJ had significantly higher average power metrics over trapezius barbell (p<0.001) CMJs (Table 1). There was a significant (p<0.001) decrease in power metrics when increasing the load, particularly when comparing the 20 kg and 60 kg CMJs in all bar modes of loading (Figure 1F).

## Discussion

The aim of the study was to evaluate the kinetics across various modes of weighted countermovement jumps. The results indicated changes in jump kinetics as weighted loads increased irrespective of the bar-type jump modes. Additionally, bar-type jump modes yielded different jump kinetic outputs.

The athletes in the current study produced the average peak force of 2460 ± 430 N. Similar results have been reported on force plates for elite level rugby players (2126±285–2726±208 N)[6, 15] and general athletes (2263±563 N) using PUSH bands.[19] As loads increase so did relative force (p=0.001–0.042). This relationship has been previously identified in other studies,[12,15,17] signifying that force needs to increase with additional load due to the increase inertia. Regarding, loaded trapezius bar jumps, similar kinetics have been reported using force plates (2353–2945 N).[6]

The participants in the current study had a peak velocity of 2.34±0.47 m.s^−1^ which was much higher than that of untrained individuals but lower than trained athletes. They did, however, show similar numbers to that of both amateur (2.10±0.14 m.s^−1^) [12] and elite rugby players (2.10±0.10 m.s^−1^).[15] Swinton et al.[6] reported similar peak velocity ranges for loaded barbell (2.28±0.17 m.s^−1^) and trapezius bar (2.39±0.18 m.s^−1^) jumps, conducted on force plates. The athletes in the study had a peak acceleration of 10.94±3.62 m.s^−2^, similar values to that of general athletes (10.65±2.24 m.s^−2^).[16] As expected and similar to previous findings,[11,12,16] significantly reduced velocities and accelerations were noted (p<0.001) as loads increased. This observation agrees with the principle of the F-v relationship which states that an increase of force will result in a decrease in velocity and as such, lighter loads will allow velocity to be greater whereas heavier loads results in greater force production at the cost of lower velocity metrics.[5] Moreover, at the muscular level, muscle tension is proportional to the inertia of the load.[3,6,17] In effect, as the overall load increases, the amount of activated muscle fibres will proportionally increase to overcome the load inertia.

The athletes of the current study produced an average power of 4477±1310 W over the nine jumping conditions (three loads and three jumping modes). The data of the current study corresponds with other elite rugby players (4509±701 W).[15] While previous research reported similar peak power outputs for barbell (4073 ± 713 W [15], 4091 ± 438 W [6]) and trapezius bar (4606 ± 510 W [6]) CMJs, the evaluation methods seem to influence the results.[6, 15] Loaded CMJ peak power outputs measured with PUSH bands ranged from 3956–4324 W, whereas concurrent force plate measurements yielded values between 3944–4073 W.[15]

Peak power in the current study (Table 1) was significantly different across the various loads, specifically between the 20 kg and 60 kg loads (p<0.001). No statistical significance was noted between the 20 kg and 40 kg loads (p=0.430), as well the 40 kg and 60 kg load (p=0.210). The power outputs of the participants specifically dropped as load increased, a result similar to previous findings,[15] which may in part be due to the drop of velocity associated with the increase in load.

Tredrea, Middleton et al.[11] reported how the positioning of an external load can affect jump kinematics and kinetics. Specifically, they reported central loads reduced propulsive phase durations compared to external loading via a barbell.[11] When assessing the loading mode of jumps, peak force was greatest in the dumbbell CMJ (2559.28±462.28 N) compared to both the trapezius barbell (2441.91±468.68 N) and barbell (1987.54±313.11 N). Trapezius barbell and dumbbell CMJs have also been found to have similar results with a consistent increase with the force metrics as loads increase,[10] which corresponds with the data of barbell CMJs. So, this trend is evident in all modes of loading regardless of the conditions.[6,12,15]

The trapezius barbell showed a significant difference in acceleration relative to the barbell and dumbbell CMJs. As expected, acceleration should influence the velocity and consequently affect the momentum of the movement.[1] As such, higher velocities were reported in trapezius barbell compared to both barbell and dumbbells. However, no differences in acceleration or velocity were noted between barbell and dumbbell CMJs. In contrast, longer flight times between dumbbell and barbell CMJs have been reported.[10] This finding may infer greater take-off velocities during dumbbell loaded jumps. In terms of power, the dumbbell CMJs produced statistically greater peak and relative power compared to trapezius barbells. While not significant, the dumbbell CMJ did consistently produce higher outputs than both the barbell and the trapezius barbell jump. This could be attributed to the added momentum caused from minor arm swings in the dumbbell CMJ.

Jump kinetics seem to be influenced by different bar-type modes of jumping. For example, the trapezius barbell CMJ may be affected by individuals swaying (movement initiated from arm swinging) compared to the other two modes of jumping.[1] Additionally, it is likely that the dumbbell produced greater forces due to the reduced arm restrictions. As an arm swing adds more momentum to a movement, it allows for a greater transfer of energy throughout the body, which in turn allows for a more powerful movement when a jump is completed.[7] Indeed, a full arm swing produces an increase of between 10.9% and 12.7% of peak force and power.[7] The reduced restrictions on arm movement when holding dumbbells could increase arm movement to about half a full swing. In theory, this could cause a 5 to 6% increase in peak metrics.[7] As the momentum of an arm swing influences the power metrics of the jumps in a positive manner, so too may the range of joint motions and jumping kinematics.[7, 11] Similarly, the various bar-type jump modes may affect the ranges of motion an athlete experiences during the jumps. That is, due to the position of the added load, athletes may be able to squat lower when loaded with trapezius barbell and dumbbells, compared to a straight barbell. Additionally, athletes might experience greater discomfort with a straight barbell compared to the other two modes, consequently reducing their preferred movement patterns.[9, 10] However, more research is required to substantiate these claims.

Previous studies have reported how loading position is beneficial to attaining peak power.[6, 9] Comparatively, power outputs are higher when loaded CMJs are performed with a trapezius barbell than a barbell.[6, 9] The greater mechanical power can be attributed to increased velocity[6, 18] and a load placement closer to the body’s CoM.[6] However, the greater mechanical power in the current study seems to be linked more to the generation of force and not velocity. The velocity data in the current study was lowest in the barbell jumps, which yielded the greatest force. Conversely, the trapezius barbell jumps produced the highest velocities and lowest forces. The evaluation mode of kinetics is likely responsible for these differences. In the current study, wearable accelerometers (PUSH 2.0) were used, compared to force plates[6] and bar-mounted linear position transducers.[18] Ideally, accelerometers should always be towards the CoM where they can measure their displacement.[19] Positioning the PUSH bands on the bars or limbs may be affected by trunk flexions and rotation and limb movements.[19] Furthermore, Orser et al.[20] have suggested that movements at faster velocities and lighter loads might be inaccurate.

The use of pre-determined flexion in the hip and knees can optimise both the power outputs and the force of the movement.[21] However, higher CMJs are recorded whenever individuals do not have an external control of flexion, which thus produces the most reliable results for testing.[21] When looking at the current study, it was observed that athletes used a similar level of flexion for all the dumbbell and barbell modes of jump but struggled to squat deeper with the trapezius barbell. This is likely due to the dimension and design of trapezius barbells. Specifically, the trapezius barbell requires the athletes to hold the bar under the waist level and may lead to the plates reaching ground level during the downward movement. It is likely that athletes limited their squat depth as a movement constraint to avoid placing the plates onto the ground. This could have resulted in reduced flexion of the hip and knees when handling the trapezius barbell.[21] During the countermovement, a lower hip and knee flexion will influence the jump's velocity and height.[21] Similarly, a reduced bar displacement when using a trapezius barbell might be a more advantageous load location for maximising power outputs.[9, 18] In their comparison between barbell and hexagonal bar jumps (HBJ) Swinton and colleagues[6] attribute jump differences in HBJ to a change in technique. Specifically, they reported that greater trunk movements in HBJ might be closer to unweighted jumps.[6]

The current study utilised accelerometers for data collection, necessitating a better understanding of their functioning. The study used the PUSH band 2.0 accelerometer, placed on the barbell and trapezius barbell during jumping protocols and around the waists for dumbbell CMJs. These devices have pre-set training modes and algorithms that likely adjust for the CoM position.[14]

While some studies have reported the PUSH band 2.0 has shown reliable results for peak force, velocity, acceleration, and power,[14] other studies have questioned their validity and reliability.[19] Wearable devices like the PUSH may lead to measurement errors if protocols and placement are not followed.[1] It is essential to consider that wearable devices are affected by body inertia, which can impact the metrics produced, particularly when arm swings are involved.[1, 19] These errors in device placement might explain why dumbbell CMJs generate more power compared to the barbell and trapezius barbell CMJs, specifically due to the added bar, trunk or arm movement during the jump attempts.[14, 19, 20]

### Practical implications

Incorporating CMJs that employ multiple modes of loading, emphasising movements with varying DoF, into an individual’s training schedule may prove beneficial in improving jump metrics and in turn power production. Specifically, understanding the kinetics of each loaded jump type can be used to guide training specificity. For example, when looking for exercises to produce the highest force unconstrained by movements, our data suggest that dumbbell CMJs would be the best option. Overall, athletes participating in sports where kinetic chain movement is constrained may benefit from barbell CMJ training. Conversely, athletes participating in sports where jump velocity is paramount would benefit from trapezius barbell CMJs.

## Conclusion

In conclusion, this study's results found that loaded CMJ kinetics change across the modes of jumping and that load placement affects the ranges of movement and comfort loading levels during the CMJs, which may be influential factors affecting loaded CMJ output metrics.

## Figures and Tables

**Table 1:** Weighted countermovement jump metrics of 32 athletes using barbell, trapezius barbell and dumbbell bar-type loading modes

Weight	Barbell	Trapezius barbell	Dumbell	Average
**Peak Force (N)**				

20 kg	2356 ± 430	2250 ± 385	2445 ± 490	2351 ± 440
40 kg	2464 ± 432	2536 ± 558	2515 ± 432	2505 ± 473
60 kg	2560 ± 418	2539 ± 397	2717 ± 432	2605 ± 419
Average	2460 ± 430	2442 ± 469	2559 ± 462	2487 ± 455

**Relative Peak Force (N.kg** **^−1^** **)**				

20 kg	27.1 ± 3.5	25.9 ± 3.1	28.0 ± 3.2	27.0± 3.4
40 kg	28.3 ± 3.3	29.1 ± 5.4	28.9 ± 2.4	28.8 ± 3.9
60 kg	29.4 ± 2.9	29.3 ± 4.1	31.3 ± 3.2	30.0 ± 3.5
Average	28.3 ± 3.4	28.1 ± 4.6	29.4 ± 3.2	28.6 ± 3.8

**Peak Velocity (m.s** **^−1^** **)**				

20 kg	2.45 ± 0.28	2.87 ± 0.35	2.51 ± 0.25	2.61 ± 0.35
40 kg	2.22 ± 0.28	2.51 ± 0.35	2.24 ± 0.26	2.32 ± 0.32
60 kg	1.99 ± 0.27	2.17 ± 0.32	2.08 ± 0.85	2.08 ± 0.55
Average	2.22 ± 0.33	2.52 ± 0.44	2.28 ± 0.56	2.34 ± 0.47

**Peak Acceleration (m.s** **^−2^** **)**				

20 kg	12.22 ± 2.70	15.45 ± 3.54	12.80 ± 2.59	13.49 ± 3.27
40 kg	9.63 ± 2.09	13.06 ± 4.78	9.86 ± 1.48	10.85 ± 3.47
60 kg	7.59 ± 1.60	9.27 ± 2.54	8.57 ± 1.59	8.48 ± 2.06
Average	9.81 ± 2.87	12.59 ± 4.49	10.41 ± 2.63	10.94 ± 3.62

**Peak Power (W)**				

20 kg	4789 ± 1285	4163 ± 873	5343 ± 1795	4765 ± 1440
40 kg	4471 ± 1118	4175 ± 1037	4849 ± 1572	4498 ± 1281
60 kg	4138 ± 1051	3974 ± 872	4392 ± 1413	4168 ± 1136
Average	4466 ± 1174	4104 ± 925	4861 ± 1632	4477 ± 1310

**Relative Peak Power (W.kg-1)**				

20 kg	55.6 ± 14.9	48.1 ± 9.3	60.4 ± 15.4	54.7 ± 14.3
40 kg	51.6 ± 12.3	48.4 ± 12.6	54.7 ± 11.8	51.6 ± 12.4
60 kg	47.5 ± 10.4	45.9 ± 9.7	49.7 ± 11.2	47.7 ± 10.5
Average	51.6 ± 13.0	47.5 ± 10.5	55.0 ± 13.5	51.3 ± 12.8

Data expressed as mean ± SD
